# Internal Polymerization of Epoxy Group of Epoxidized Natural Rubber by Ferric Chloride and Formation of Strong Network Structure

**DOI:** 10.3390/polym13234145

**Published:** 2021-11-27

**Authors:** Kriengsak Damampai, Skulrat Pichaiyut, Subhradeep Mandal, Sven Wießner, Amit Das, Charoen Nakason

**Affiliations:** 1 Faculty of Science and Industrial Technology, Surat Thani Campus, Prince of Songkla University, Surat Thani 84000, Thailand; gotzasayno@gmail.com (K.D.); skulrat.p@psu.ac.th (S.P.); 2 Leibniz-Institut für Polymerforschung Dresden e.V., D-01069 Dresden, Germany; mandal@ipfdd.de (S.M.); wiessner@ipfdd.de (S.W.); das@ipfdd.de (A.D.); 3 Institute of Materials Science, Technische Universität Dresden, D-01062 Dresden, Germany

**Keywords:** epoxidized natural rubber, internal polymerization, ferric chloride, metal ion crosslinkers, crosslinking density

## Abstract

In this work, studies are carried out to understand the crosslinking reaction of epoxidized natural rubber (50 mol% epoxy, ENR-50) by metal ion namely ferric ion (Fe^3+^, FeCl_3_, ferric chloride). It is found that a small amount of FeCl_3_ can cure ENR to a considerable extent. A direct interaction of the ferric ion with the epoxy group as well as internal polymerization enable the ENR to be cured in an efficient manner. It was also found that with the increased concentration of FeCl_3_, the crosslinking density of the matrix increased and therefore, the ENR offers higher mechanical properties (i.e., modulus and tensile strength). In addition, the glass transition temperature (*t_g_*) of ENR vulcanizate is increased with increasing concentration of FeCl_3_. Moreover, the thermal degradation temperature (T_d_) of the ENR-FeCl_3_ compound was shifted toward higher temperature as increasing concentration FeCl_3_.

## 1. Introduction

Vulcanization of rubber is a process to form permanent rubber network structures with useful properties. Typically, vulcanization is performed by sulfur vulcanization process to form sulfidic linkages between rubber chains. The presence of unsaturation in the chemical structure of rubber macromolecules enables this crosslinking process. The examples of such systems are styrene butadiene rubber (SBR) [1], natural rubber [2,3,4], and epoxidized natural rubber (ENR) [5,6,7]. Typically, the sulfur vulcanization provides the sulfidic linkages with mono-, di-, and poly-sulfidic bonds in the rubber vulcanizates [8]. The sulfur cured vulcanizates generally exhibit good mechanical and other technical properties, but the chemistry behind of such crosslinking is rather complex. It consists of at least three different chemical ingredients including activator, accelerator and curing agent. Other commonly used vulcanization systems of rubber are peroxide, phenolic and metal ions [9].

Incorporation of metal ions in epoxidized natural rubber (ENR) can lead to crosslinking reaction, and it is supposed that the network formation may be associated with coordination crosslinks (such as, Fe^3+^–O bond) [9]. However, the detail study with ENR and the metal ion cured system is not well understood. On the other hand, reaction between metal ions and other polymers (non-rubber) have been widely investigated, for instance, epoxy resin cured with ammonium ionic liquids and palladium complex catalyst [10]. A few reports can also be found where three different divalent metal ions (i.e., Ca^2+^, Cu^2+^ and Fe^2+^) are mixed with ENR and it was found that the curing characteristics and dynamic properties of ENR compounds with respect to scorch and cure times, cure rate index, storage modulus and damping peaks [11] are largely altered. It was also found that the Lewis acid characteristic of metal ions caused significant influence on the vulcanization process [11]. In addition, the transition metal ions (i.e., Co^2+^, Cu^2+^, Mn^2+^, Fe^2+^, Ni^2+^ and Ag^+1^) promoted oxidative degradation of natural rubber molecules, causing the predominant chain scission reaction during storage of dry rubber [12]. Furthermore, the effect of divalent metal ions (i.e., Ca^2+^, Sr^2+^, Mg^2+^, Cu^2+^, Zn^2+^) on intrinsic viscosity of natural rubber from Manihot glaziovii was investigated, and it was found that the intrinsic viscosity of natural rubber latex depends on the type and concentration of divalent metal ions [12]. Additionally, the thermal behavior and non-isothermal degradation of magnetite (Fe_3_O_4_)/epoxidized natural rubber (ENR-50) composites, lithium triflate (LiCF_3_SO_3_)-ENR-50 polymer electrolytes (PEs) and LiCF_3_SO_3_-Fe_3_O_4_/ENR-50 composite polymer electrolytes (CPEs) are investigated using differential scanning calorimetry (DSC) and thermogravimetric analysis (TGA) [13]. It was found that the glass transition temperature (*t_g_*) and other related thermal properties of these materials generally follow the increasing trend [13]. Recently, Zn^2+^ was incorporated in butadiene−styrene−vinylpyridine rubber (VPR) to construct additional Zn^2+^-pyridine coordination in rubber network and to tailor the mechanical performance [14]. Other metal ions (i.e., Ni^2+^, Co^2+^, Fe^3+^, La^2+^) are also incorporated into VPR at the stoichiometric equivalence of pyridine and metal ion [14]. It was found that the metal–ligand bonds are formed through the coordination reaction between the pyridine groups in butadiene–styrene–vinylpyridine rubber (VPR) and metal ions. This contributes to increase the stiffness of the material at small strain and rupture and with increasing strain, a complete recovery was achieved after stretching [14]. Additionally, strong and tough elastomers have been developed by introduction of deformable microphase-separated granules with rich epoxy–Fe^3+^ coordination into a ductile rubber network of epoxidized natural rubber [15]. It was found that the rigid granule was served as additional crosslinks to increase the modulus and toughness of the material while maintaining stretchability and recoverability [15].

In the present work, epoxidized natural rubber with 50 mol% epoxide (ENR-50) was treated with very small amount of FeCl_3_ ranging from 1 mmol to 10 mmol and the compounds were systematically studied to understand the mode of curing and related mechanisms. The crosslinked materials were compared with conventional sulfur vulcanizates in terms of mechanical and dynamic mechanical properties. Studies like stress-relaxation and dielectric relaxation spectra are done to enlighten the network structure provided by ferric chloride.

## 2. Materials and Methods

### 2.1. Materials

Materials used in this work and their sources are listed in Table 1.

### 2.2. Preparation of ENR-FeCl_3_ Compounds

ENR-50 is first dried in a hot air oven at 60 °C for about 24 h and then mixed with different concentrations of ferric chloride (1, 3, 5, 7 and 10 mmol) using an internal mixer (Brabender Plasticorder, 50 EHT, Duisburg, Germany). The initial temperature of the mixing was 60 °C and a rotor speed was 60 rpm. ENR is masticated for about 3 min before addition of ferric chloride in the mixing chamber and the mixing is continued for 8 min. ENR compounded with the conventional sulfur vulcanization (CV) system are also prepared for the comparison purpose. The ENR compounded with CV cured system is prepared by mixing ENR-50 with various chemical ingredients, as shown in Table 2. This was done by masticating of ENR-50 about 3 min and then activators, (i.e., zinc oxide and stearic acid), cure accelerator (MBTs) and curing agent (sulfur) are sequentially added. The rubber compound was then dumped from the mixing chamber and sheeted out by passing through the 1 mm nip of an open two-rolls mill model YFCR 600, Yong Fong machinery Co., Ltd. (Samut Sakorn, Thailand) at ambient temperature. The rubber compound was then conditioned in a desiccator at room temperature for about 24 h. Cure characteristics were eventually investigated by moving die rheometer, MDR 2000, Alpha-technologies, (Hudson, OH, USA) at 160 °C. Finally, the vulcanized rubber sheets are prepared by compression molding using PR1D-W400L450PM molding machine, Charon Tut Co, Ltd. (Bangkok, Thailand) at 160 °C maintaining the respective cure times based on the rheometer test.

### 2.3. Cure Characterization

Cure characteristics of various rubber compounds are investigated by moving die rheometer, MDR 2000, Alpha Technologies, (Hudson, OH, USA) with a fixed frequency of 1.67 Hz and strain amplitude of 1° arc at 160 °C. The scorch time (t_s1_), cure time (t_c90_), minimum torque (M_L_), maximum torque (M_H_), and torque difference (M_H_-M_L_), are determined from the curing curves.

### 2.4. Morphological Properties

Morphological properties and elemental composition of various ENR-50 compounds are investigated by a scanning electron microscope, SEM/EDX, model Zeiss, Supra–40 VP, Carl Zeiss Microscopy GmbH (Oberkochen, Germany). The rubber vulcanizates are first cryogenic cracked in liquid nitrogen and then gold coated before SEM characterization.

### 2.5. Fourier Transform Infrared (FTIR) Spectroscopy

The FTIR spectra of various ENR-50 compounds are recorded by transmission mode of the Fourier Transform Infrared Spectrophotometer, model Vertex70, Bruker, (Karlsruhe, Germany). Thin sheets of the neat ENR-50, ENR-50 compounded with FeCl_3_ and with the CV cured system are first fabricated and then characterized by attenuated total reflection (ATR) FTIR. The analysis was performed over the wide wavenumber ranges from 4000 to 400 cm^−1^.

### 2.6. Mechanical Properties

The tensile test specimens are mechanically die cut from the rubber vulcanizate sheets according to ISO 527 (type 5A). The samples were then clamped with the sample holder of the tensile testing machine, Zwick GmbH & Co., KG (Ulm, Germany) at room temperature. They are then elongated with a crosshead speed of 200 mm/min, according to ISO 527. The hardness of various rubber vulcanizates was also determined by durometer shore A, Instron, (Norwood, MA, USA), according to ISO 868.

### 2.7. Crosslink Density

Swelling experiments are carried out to determine the crosslink density of the rectangular 10 × 10 × 2 mm^3^ rubber specimens. The weight of the rubber samples is taken before immersing them into toluene at room temperature for seven days. The swollen rubber samples are then removed and excess liquid on the specimen surfaces was wiped out by a filter paper. After that, the specimens are dried in a vacuum oven at 40 °C for 24 h. Finally, the original weight was compared with the final weight before and after immersion into toluene. The crosslink density of the rubber vulcanizates was eventually determined according to Flory–Rehner relation [16]:

(1) V=−In(1−∅p)+∅p+Ꭓ · ∅p2VL·(∅p13−∅p2)

where ∅ is the volume fraction of rubber in the swollen network, $V_{L}$ is the molar volume of toluene and *Ꭓ* is the interaction parameter of polymer and solvent (for ENR and toluene, the value is 0.34) [16].

### 2.8. Thermo Gravimetric Analysis (TGA)

Thermal stability of neat ENR-50, ENR-50 crosslinked with sulfur and FeCl_3_ cured ENR examined by thermogravimetric analysis by using TGA-SDTA 851, Mettler Toledo (Zurich, Switzerland). The measurement is performed under nitrogen atmosphere in the temperature ranges 30–600 °C before switching to oxygen atmosphere with the same heating rate (i.e., 10 °C/min) until 900 °C.

### 2.9. Temperature Scanning Stress Relaxation

The temperature scanning stress relaxation (TSSR) measurements of rubber vulcanizates are performed using a TSSR meter, Brabender Messtechnik^®^ GmbH & Co. KG, (Duisburg, Germany). Dumbbell-shaped specimens (type 5A) according to ISO 527 are first annealed in a hot air oven at 100 °C for 30 min to eliminate thermal history of NR. They are then cooled down to room temperature for about 30 min before further TSSR tests. The sample is then stretched up to 50% of its original length at 23 °C and then conditioned at this temperature for about 2 h. After that, the non-isothermal test is applied by raising the temperature from 23 °C to 220 °C with a constant heating rate of 2 °C/min (v). The stress at each temperature is then recorded and reported in terms of relationship between stress and tested temperature and hence relaxation modulus and temperature. It is noted that the relaxation spectrum (H(T)) was also calculated based on the relationship between relaxation modulus (E(T)) and temperature (T), as follows [17]:

(2) $$H\left(T\right)=-T{\left[\frac{dE\left(T\right)}{dT}\right]}_{\nu =const}\;$$

### 2.10. Dynamic Mechanical Analysis

Dynamic mechanical analysis (DMA) is carried out using DMA 8000, Perkin Elmer Inc. (Waltham, MA, USA) in a tension mode in the temperature ranges from −100 to 80 °C with a heating rate of 3 °C/min and a fixed deformation frequency of 1 Hz.

### 2.11. Electrical Properties

Electrical properties in terms of electrical conductivity (σ) and dielectric constant (ε′) of the neat ENR-50, sulfur cured ENR-50 and FeCl_3_ cured ENR are measured at room temperature using an LCR meter, model IM 3533, Hioki E.E. Corporation (Nagano, Japan) in the frequency ranges of 1 to 105 Hz. It is noted that the LCR meter is connected to the electrode plates of a dielectric test fixture model 16451B dielectric test fixture, Test Equipment Solutions Ltd. (Berkshire, UK), with 5 mm electrode diameter. The electrical conductivity (σ) and dielectric constant (ε′) are determined, as following relations [18,19]:

(3) $$\sigma =\frac{1}{\rho }=\frac{d}{\left(R_{p}\right)A}$$

(4) $$\varepsilon^{\prime }=\frac{C_{p}\left(d\right)}{A\left(\varepsilon_{0}\right)}\;$$

where *d* and *A* refer to the sample thickness and the area of an electrode, respectively. The parameter $\varepsilon_{0}$ is the dielectric constant of the free space, which is 8.854 × 10^−12^ F/m. The factor ρ is the volume resistivity, which is reciprocal of conductivity.

## 3. Results and Discussion

### 3.1. Curing Characteristics

**Figure 1:**
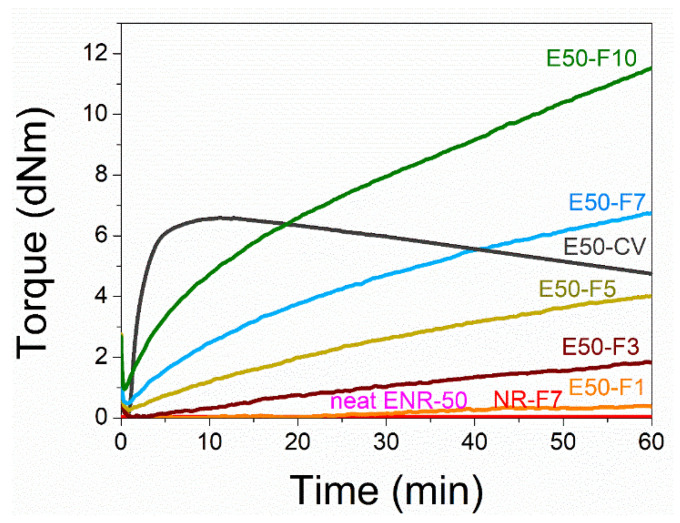
Mixing torque-time curves of ENR-50 compounds with various concentrations of FeCl_3_ at 1, 3, 5, 7 and 10 mmol (i.e., E50-F1, E50-F3, E50-F5, E50-F7 and E50-F10, respectively), and the conventional sulfur vulcanization (i.e., E50-CV), neat ENR-50, and unmodified NR (ADS) with 7 mmol of FeCl_3_ (NR-F7).

**Figure 2:**
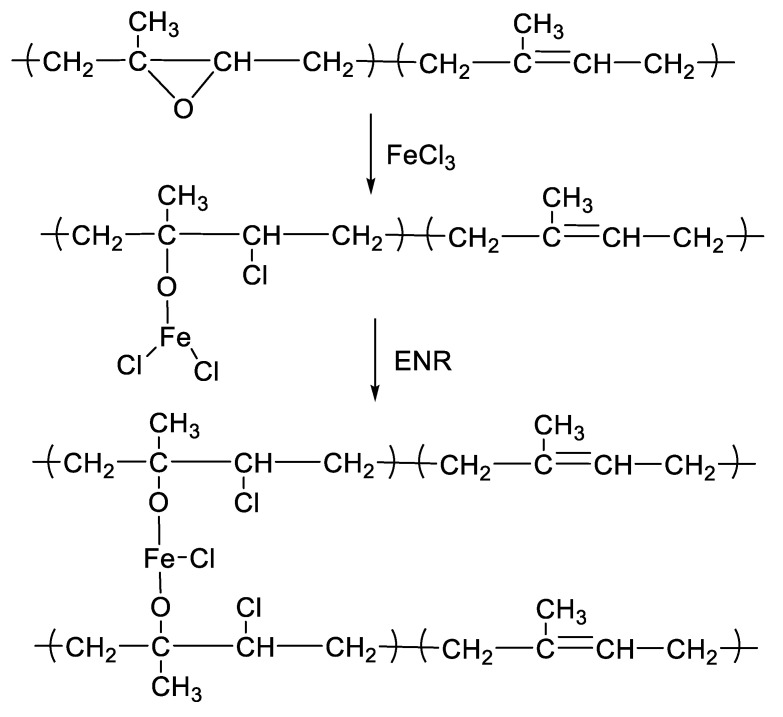
A proposed reaction mechanism between epoxidized natural rubber and ferric chloride. The nucleophilic chloride is attached with β position with respect to the methyl group.

**Figure 3:**
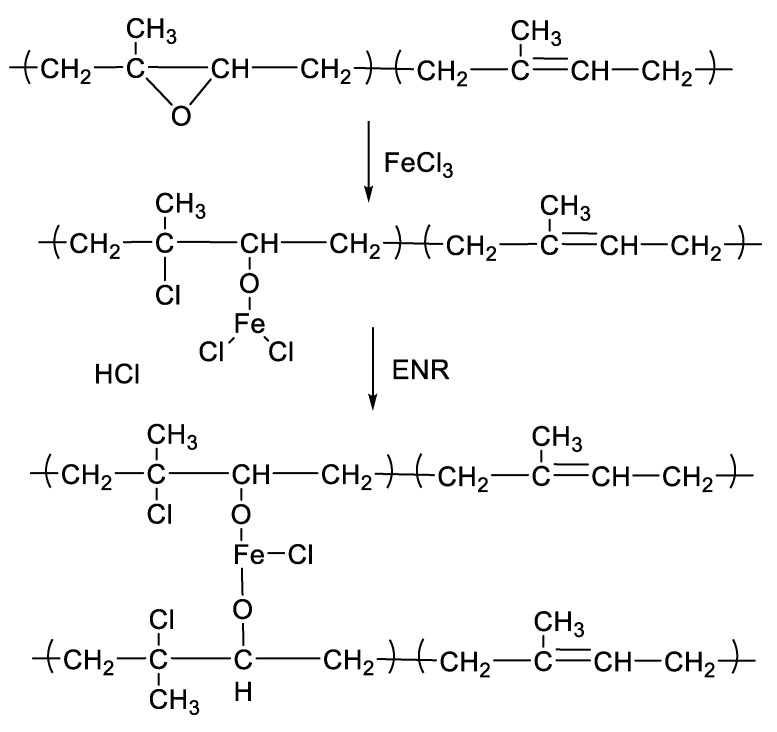
A proposed reaction mechanism between epoxidized natural rubber and ferric chloride. The nucleophilic chloride is attached with β position with respect to the methyl group.

**Figure 4:**
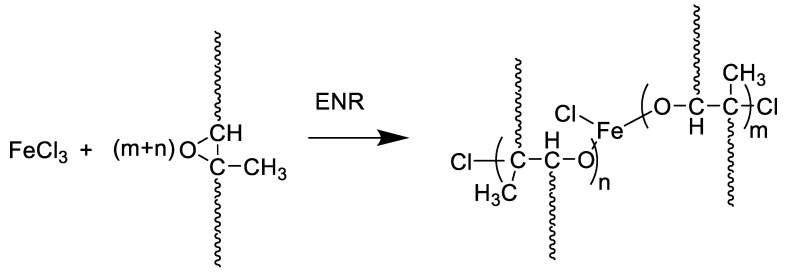
A proposed reaction mechanism between epoxidized natural rubber and ferric chloride. In the mechanism, the ring opening type polymerization of the epoxy group is described, which can be correlated with “internal polymerization” of the epoxy group of epoxide natural rubber.

Figure 1 shows the mixing torque-time curves of the ENR-50 compounded with various concentrations of FeCl_3_ (1, 3, 5, 7 and 10 mmol). The conventional sulfur compound (i.e., E50-CV) is also considered for comparison. It is clearly seen that the neat ENR-50 and unmodified NR (ADS) with 7 mmol FeCl_3_ had no response of mixing torque-time relation, indicating no establishment of network structure. However, slightly increasing torque is obvious when ENR-50 was mixed with 1 mmol of FeCl_3_ (E50-F1). Furthermore, increasing concentrations of FeCl_3_ from 1 to 10 mmol caused abruptly increasing of torque at a given testing time in particularly, at high loadings of FeCl_3_. Additionally, the steepness of the mixing torque-time curves is increased with increasing concentrations of FeCl_3_ resulting a marching nature of the crosslinking reaction. The curing curves of ENR- FeCl_3_ compounds have not reached to the equilibrium curing state within the experimental time of 1 h. Table 3 shows cure characteristics in terms of minimum torque (M_L_), maximum torque (M_H_), torque difference (M_H_-M_L_), scorch time (t_s2_) and cure time (t_c90_) of various ENR-50 compounds. It is seen that the maximum rheometric torque is increased with increasing FeCl_3_ concentration. This might be attributed to chemical reaction between oxirane rings of the ENR molecules and Fe^3+^ ion to form crosslinking network structures via -O-Fe-O- linkages. The proposed reaction mechanism is shown in Figure 2, Figure 3 and Figure 4. It is noted that under the given conditions at high temperature, the oxirane rings in ENR molecules are prone to ring opening reaction with a product that are able to form new linkages with another ring opened ENR fragment via Fe^3+^ bridge. In Figure 2, Figure 3 and Figure 4, different types of crosslinking structures are described [20,21]. As far as the chemical scheme of Figure 2 and Figure 3 are concerned, the microstructure in Figure 3 is more favorable due to presence of methyl group that enables the adjacent carbon atom less nucleophilic. It could enhance the possibility of nucleophilic attack by chloride ion to the carbon atom without the methyl group. Furthermore, the strong reactivity of ethylene oxide or propylene oxide with ferric chloride is very well-known and is described very well [20]. It is described that the epoxy group containing molecules can even undergo a “internal polymerization” type of reaction and yield many exchanged and complicated polymeric microstructures. In our present case, the epoxy groups are most probably following similar types of reaction pathways and can result in very strong crosslinking structures as described in Figure 4. Due to the remarkable reactive character of ferric chloride, the epoxy group can follow a ring opening type “internal polymerization” resulting highly dense network structure. In Figure 1, it is also seen that the ENR-50 compounded with conventional sulfur vulcanization system (ENR-CV) exhibits a typical curing curve with dramatically reversion phenomenon at the curing time beyond 10 min. This is due to the destruction of newly formed sulfidic linkages due to high temperature treatment. In Table 3, it is also clear that the ENR-50 compounded with FeCl_3_ over 7 mmol showed higher maximum rheometric torque and curing time than the E50-CV. This may reflect a higher content of crosslink density. It is also clear that increasing concentration of ferric chloride caused a decrement of the cure rate index (CRI). This may be attributed to the cure retarding effect of chloride ion of FeCl_3_ (Figure 1).

### 3.2. Attenuated Total Reflection (ATR) Fourier Transform Infrared Spectrophotometer (FTIR)

**Figure 5:**
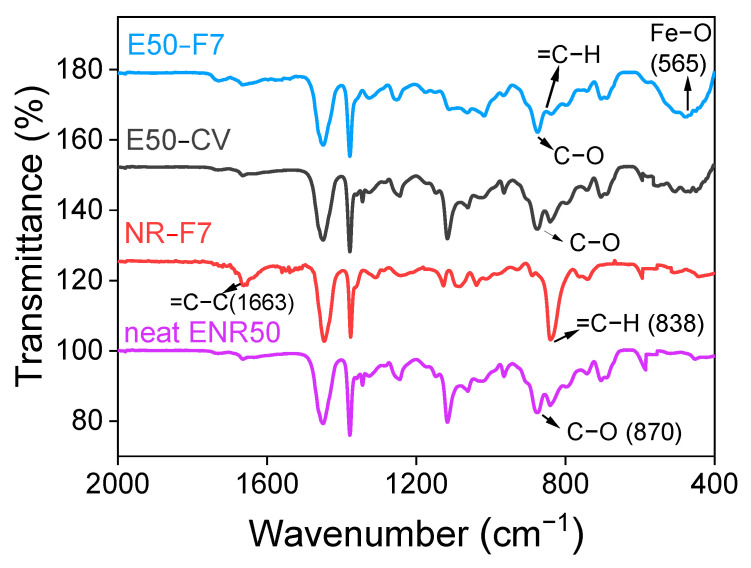
ATR-FTIR spectrum of the neat ENR-50, ENR-50 compounded with 7 mmol of FeCl_3_ (E50-F7) and the conventional sulfur vulcanization (E50-CV) as well as the unmodified NR (ADS) compounded with 7 mmol FeCl_3_ (NR-F7).

Figure 5 shows ATR-FTIR spectra of neat ENR-50, ENR-50 compounded with 7 mmol of FeCl_3_ (E50-F7), and conventional sulfur vulcanizates (E50-CV), together with unmodified NR (ADS) compounded with 7 mmol FeCl_3_ (NR-F7). It is clear that the FTIR absorption peak at the wavenumber 878 cm^−1^ is clearly seen in the FTIR spectrum of the neat ENR-50, which indicates the presence of the asymmetric C–O stretching vibration in epoxide rings of ENR molecules [22]. Additionally, the main absorption peaks at the wavenumbers 878 cm^−1^ and 838 cm^−1^ are also observed in the FTIR spectra of ENR-FeCl_3_. They assign to the asymmetric C–O stretching vibration in oxirane rings and =C–H out of plane bending in ENR molecules [23]. However, a broad new absorption peak at wavenumber of 470 cm^−1^ is clearly observed in the FTIR spectra of ENR-FeCl_3_. However, this broad peak may be consisting with many other peaks more particularly, at 544 and 444 cm^−1^, which are well identified as Fe–O bond in the structure of α-Fe_2_O_3_ [24]. This indicates the existence of the Fe–O stretching vibration in ENR-FeCl_3_ compounds [23]. Therefore, the newly formed linkages between ENR and FeCl_3_ molecules is responsible for the absorption peak at wavenumber 470 cm^−1^. In Figure 5, it is also clear that incorporation of 7 mmol FeCl_3_ in ENR-50 caused a reduction of the peak intensity at wavenumber 838 cm^−1^, which might represent the disappearance of some oxirane rings.

### 3.3. Mechanical Properties

**Figure 6:**
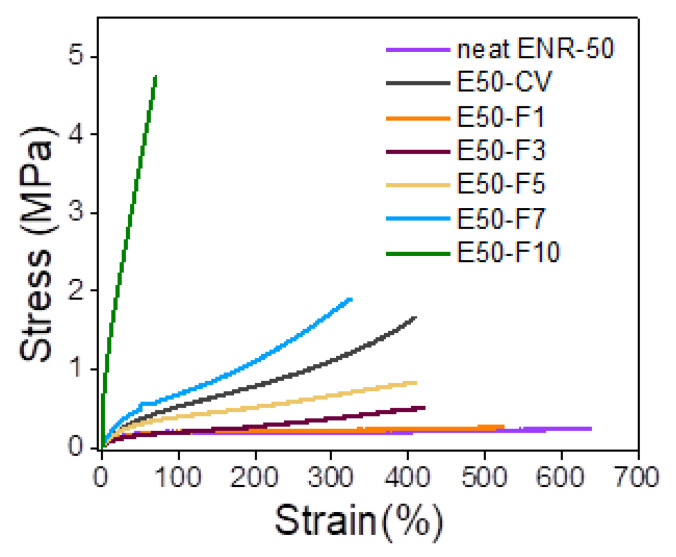
Stress–strain curves of ENR-50 compounded with various concentrations of FeCl_3_ at 1, 3, 5, 7 and 10 mmol, and the conventional sulfur vulcanization (i.e., E50-CV).

Figure 6 shows stress–strain curves of ENR-50 compounded with various concentrations of FeCl_3_ and the data are compared with conventional sulfur vulcanizate (i.e., E50-CV). Additionally, mechanical properties in terms of 100% modulus, tensile strength, elongation at break and hardness (Shore A) of various ENR-50 compounds are summarized in Table 4. It is seen that ENR-50 compounded with FeCl_3_ offered significantly stronger initial modulus (Young’s moduli) (i.e., slope of the initial curves) with increasing concentrations of FeCl_3_. Additionally, the toughness as indicated by the area underneath of the stress–strain curve (Table 4) also increased with increasing concentrations of FeCl_3_. This is attributed to the increasing level of crosslinking reaction and hence the linkages between oxirane rings in ENR molecules and FeCl_3_ to form more rubber network structures due to internal polymerization as shown in Figure 4. In Table 4, it can be observed that a lower 100% modulus and tensile strength of ENR-50 compounded with low concentration of FeCl_3_ (i.e., 3 and 5 mmol) as compared with the ENR-50 compounded with the conventional sulfur vulcanization. However, with the increasing concentration of FeCl_3_ in ENR-50 to 7 and 10 mmol, 100% modulus and tensile strength are increased as compared with the data obtained from E50-CV. This might be due to higher contents of the crosslinking structures by internal polymerization. On the other hand, the elongation at break decreased with increasing the FeCl_3_ contents (Table 4). This might be attributed to the restriction of the rubber chain mobility of ENR molecules caused by increasing concentrations of FeCl_3_, which provided higher contents of strong rubbery network with higher crosslinking density. In Table 4, it is also clear that hardness of the ENR-50 compounds with FeCl_3_ increased with increasing concentrations of FeCl_3_. This can be explained by increasing contents of –O–Fe–O linkages between ENR-50 molecules to cause higher level of crosslinking density. In addition, the ENR-50 compounds with higher amounts of FeCl_3_ show hardness values that are higher than the E50-CV. This confirms a denser rigid crosslinking network. This observation is also reflected in the maximum rheometric torque values (Table 3). In Figure 6, it is also seen that the stress–strain curve of E50-F10 exhibits a rigid solid-like behavior with the highest hardness (Table 4) and brittle properties, as indicated by high moduli, but less elongation at break (Figure 6).

### 3.4. Crosslinking Density

Table 5 shows the crosslinking densities of ENR-50 compounded with various concentrations of FeCl_3_ at 1, 3, 5, 7 and 10 mmol and the conventional sulfur vulcanizates (i.e., E50-CV). It is clearly seen that the crosslinking densities of ENR-50 compounded with FeCl_3_ over 7 mmol are higher than the E50-CV. In addition, the crosslinking densities increased with increasing FeCl_3_ contents due to the higher crosslinks between Fe^3+^ and oxygen of the oxirane rings as well as the impact of the internal polymerization. Moreover, E50-F7 and E50-F10 show higher crosslinking density than E50-CV. This indicates higher level of chemical crosslinks between ENR molecules and hence higher contents of -O-Fe-O linkage in the elastomer networks. This result correlates with the value of maximum rheometric torque in Table 3.

### 3.5. Thermogravimetric Analysis

**Figure 7:**
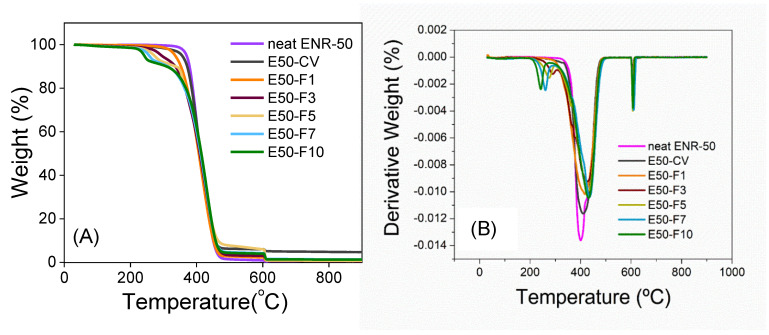
TGA (**A**) and DTG thermograms (**B**) of neat ENR-50 and ENR-50 compounded with various concentrations of FeCl3 at 1, 3, 5, 7 and 10 mmol the conventional sulfur vulcanization system (i.e., E50-CV).

Figure 7 shows the TGA and DTG thermograms of the neat ENR-50 and ENR-50 compounded with various concentrations of FeCl_3_ and the conventional sulfur vulcanization system (i.e., E50-CV). It is noted that TGA was performed under nitrogen atmosphere in the temperature ranges between 23–600 °C, before switching to oxygen atmosphere and heated until 900 °C with the same heating rate at 10 °C/min. The TGA thermograms of ENR-50 in Figure 7A, compounded with FeCl_3_ show double degradation steps in the temperature ranges around 240–290 °C and 418–433 °C. The first DTG peaks (Figure 7B) at around 240–290 °C (T_d1_ in Table 6) can be associated with dissociation of water molecules in the crystal structure of FeCl_3._ The second DTG peaks are seen around 418–433 °C (Td2 in Table 6), indicating the degradation of hydrocarbon in ENR molecules. In addition, an increase in concentrations of FeCl_3_ causes shifting of the degradation temperature of ENR molecules (i.e., T_d2_) to higher temperature (Table 6). This might be due to higher thermal resistance of the material with –O-Fe-O- linkages. On the other hand, the degradation of the neat ENR-50 and E50-CV showed a single degradation stage with no significant difference in the TGA thermograms with the DTG peaks (i.e., decomposition temperature (T_d_)) around 402 and 411 °C, respectively. This is attributed to the degradation of ENR molecules. Nevertheless, in the last region under the oxygen atmosphere of TGA thermogram (Figure 7A and Table 6), the ENR50-CV exhibited the highest remained residue, as compared to the ENR-50 compounded with FeCl_3_ and the neat ENR-50, respectively. This might be more particulate chemicals involved in the conventional sulfur vulcanization system such as ZnO.

### 3.6. Temperature Scanning Stress Relaxation (TSSR)

**Figure 8:**
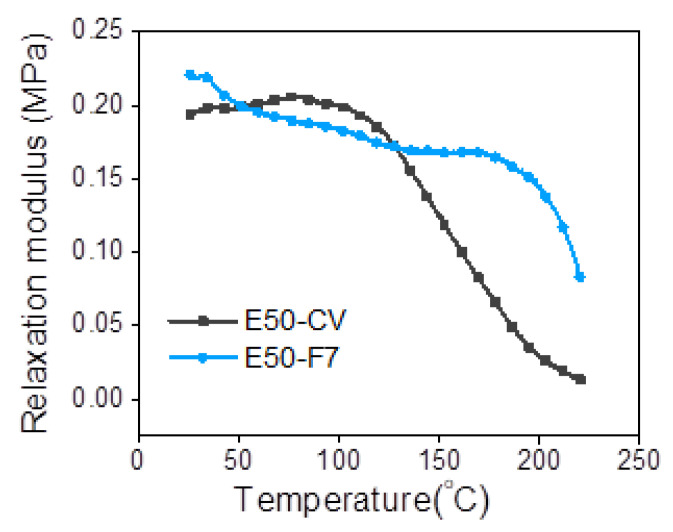
Representative relaxation modulus of ENR-50 compounded with 7 mmol of FeCl_3_ (E50-F7) and the conventional sulfur vulcanizate (i.e., E50-CV).

Figure 8 shows the relaxation modulus as a function of temperature of ENR compounded with 7 mmol of FeCl_3_ (E50-F7) and the conventional sulfur vulcanizate (E50-CV). It can be seen that the E50-F7 shows higher initial modulus than the E50-CV. This might be due to the higher level of crosslink structures. This can be corelated with the data of delta torque (Table 3), crosslinking density (Table 5) and the 100% modulus (Table 4). In Figure 8, it is also seen that that the initial relaxation modulus of E50-F7 is marginally increased, but E50-CV is remarkably increased with increasing temperature in the initial region. This might be contributed to the entropy effect, which typically happens in the rubber vulcanizates [25]. Another part of the modulus curves in the temperature ranges from 30 to 90 °C indicated the deterioration of the physically bound rubber molecules. Furthermore, the thermal degradation of sulfidic crosslinks and rubber chains are observed in the temperature ranges between 90–160 °C and 160–220 °C, respectively [25]. In Figure 8, it is clear that the E50-F7 has a higher entropy effect than the sulfur crosslinked sample, E50-EV. Additionally, the relaxation modulus of E50-CV showed rapidly decreasing to nearly zero in the temperature ranges 80–220 °C. This is attributed to lower thermal resistance of the E50-CV than E50-F7. This result correlates to lower degradation temperature (T_d_) of E50-CV than E50-F7 in Table 6.

### 3.7. Dynamic Properties

**Figure 9:**
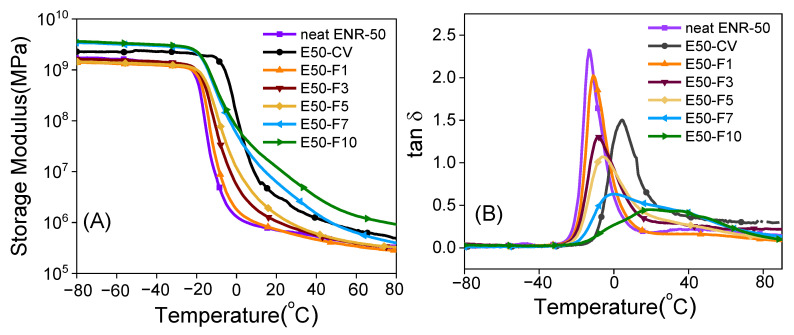
Storage modulus (**A**) and tan δ (**B**) as a function of temperature of the neat ENR-50, ENR-50 compounded with various concentrations of FeCl_3_ compared and the conventional sulfur vulcanizate (i.e., E50-CV).

Figure 9 shows the storage modulus (E′) and the loss tangent (tan δ) as a function of temperature of the neat ENR-50 and ENR-50 compounded with various concentrations of FeCl_3_ and with the conventional sulfur vulcanization (i.e., E50-CV). In Figure 9A, it is clearly seen that the storage moduli in the glassy regions of E50-F7 and E50-F10 are higher than the E50-CV and other types of materials investigated. This might be due to formation of the stronger rubber networks that contain high amount of -O-Fe-O- linkages in the E50-F7 and E50-F10 vulcanizates. In the ENR-50 with lower FeCl_3_ concentration than 7 mmol, it is clear that lower and weaker molecular networks of ENR are formed. This might be due to the lower extent of crosslinking reactions and hence lower new formed -O-Fe-O- coordination linkages. In Figure 9A, it is seen that increasing temperature approaches the glass transition zone where the glass transition temperature (Tg) of various materials is seen, as the tan δ peaks in Figure 9B. It is clear that increasing concentration of FeCl_3_ in ENR-50 compounds caused higher glass transition temperature (*t_g_*). This may be associated with the increase in the crosslinking densities (Table 5) with more restriction in the rubber chain mobility. It is also clear that the ENR compounds with FeCl_3_ indicate only a single glass transition temperature (*t_g_*), which is seen from the single tan δ peak in Figure 9B. In Figure 9B, it is clearly seen that the ENR-FeCl_3_ compounds with FeCl_3_ greater than 3 mmol had lower tan δ peak height. This is attributed to lower level of chain mobility of the materials with higher crosslinking density and hence higher glass transition temperature (*t_g_*). In the rubbery region, E50-F7 and E50-F10 showed higher storage moduli than the E50-CV and other types of materials (Figure 9A). This is due to the higher crosslinking density based on strong reactions between FeCl_3_ and the epoxide groups of the ENR molecules.

### 3.8. Electrical Properties

**Figure 10:**
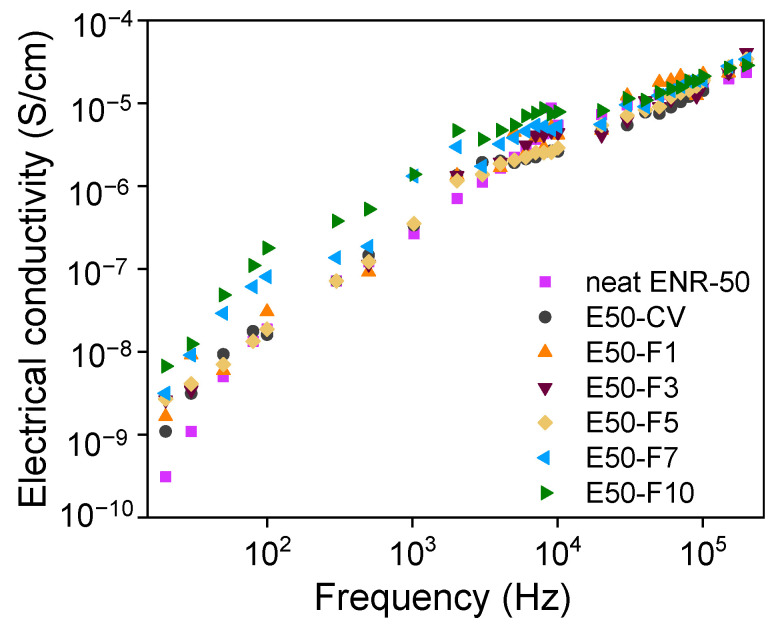
Electrical conductivity as a function of frequency of the neat ENR-50, ENR-50 compounded with various concentrations of FeCl_3_ and the conventional sulfur vulcanizate (i.e., E50-CV).

Figure 10 shows electrical conductivities of ENR compounds as a function of frequencies. It seen that the neat ENR and E50-CV show lower electrical conductivity.

However, upon addition of various concentrations of FeCl_3_ in ENR-50, suddenly improvement of the electrical conductivity of the ENR-FeCl_3_ compounds is clearly seen. Additionally, the electrical conductivities increased with increasing concentrations of FeCl_3_ in ENR-50 with strong increment with the concentration of FeCl_3_ beyond 5 mmol. This might be due to higher electrical conductivity of FeCl_3_, causing powerful transferring of electrical currents and enhances the conductivity of rubber compound significantly.

## 4. Conclusions

ENR-50 can be very efficiently crosslinked by using FeCl_3_. In the present case, a small amount of FeCl_3_ was used to cure ENR. The linkages between FeCl_3_ and oxirane ring in the ENR molecule (i.e., -O-Fe-O) was confirmed from the FTIR studies. It was found that an increased concentration of FeCl_3_ improved the properties of ENR-50 compounds, including maximum rheometric torque, crosslinking densities, tensile strength, dynamic properties, electrical conductivity and thermal properties. The conventional sulfur vulcanization of ENR-50 compound and unmodified NR compounded with 7 mmol FeCl_3_ were used for comparison and it is found that FeCl_3_ can not cure NR. Furthermore, the ENR-50 compounded with FeCl_3_ at 7 and 10 mmol showed higher crosslinking densities and mechanical properties than the ENR-50 cured with conventional sulfur. This might be due to higher crosslinking structures of the cured samples. Additionally, the internal polymerization of epoxy moieties with FeCl_3_ offers very dense network structures, which are reflected in mechanical and dynamic mechanical properties. Additionally, *t_g_* values increased with increasing concentrations of FeCl_3_. This is due to the increase in the crosslinking densities that restricted the mobility of ENR molecules. Furthermore, the ENR-50 compounded with FeCl_3_ showed the highest electrical conductivity than other samples without FeCl_3_. Additionally, thermal resistance was increased with increasing concentration of FeCl_3_. The reaction between ENR and FeCl_3_ could be very rapid at a higher temperature and the principle can be applied for other epoxy-based materials.

## Figures and Tables

**Table 1:** Characteristics and sources of materials used.

No.	Chemicals	Sources
1	Epoxidized natural rubber with 50 mol% epoxide (ENR-50)	Muangmai Guthrie Co. Co., Ltd., (Surat Thani, Thailand)
2	Natural rubber (NR), Air dry sheet (ADS)	Von Bundit Co., Ltd. (Surat Thani, Thailand)
3	Ferric chloride (FeCl_3_)	Sigma-aldrich, (St. Louis, MO, USA)
4	Sulfur (S)	Ajax Chemical Co., Ltd., (Samut Prakan, Thailand)
5	Zinc oxide (ZnO)	Global Chemical Co., Ltd., (Samut Prakarn, Thailand)
6	Stearic acid	Imperial Industry Chemical Co., Ltd., (Pathum Thani, Thailand)
7	2,2-Dithiobis-(benzothiazole) (MBTs)	Flexsys, (Termoli, Italy)

**Table 2:** Formulations of the elastomer composites.

Sample No. *	ENR-50 (g)	FeCl_3_ (mmol)	Abbreviation
1	100	0	neat ENR-50
2	100	1	E50-F1
3	100	3	E50-F3
4	100	5	E50-F5
5	100	7	E50-F7
6	100	10	E50-F10

* Two controlled batches are prepared: 100 g ENR-50, 5g ZnO, 1 g stearic acid, 0.5 g MBTs, 2.5 g sulfur. This is designated as E50-CV. In another one, 100 g NR is mixed with 7 g of FeCl_3_ and it is designated as NR-F7.

**Table 3:** Cure characteristics in terms of minimum torque (M_L_), maximum torque (M_H_), torque difference (M_H_-M_L_), scorch time (T_s2_) and cure time (T_c90_) of various ENR-50 compounds.

Compounds	M_L_ (dN.m)	M_H_ (dN.m)	M_H_-M_L_ (dN.m)	T_s2_ (min)	T_c90_ (min)	CRI
E50-CV	0.22	6.18	5.96	2.11	7.36	19.04
E50-F1	0.12	0.47	0.35	3.75	6.40	37.73
E50-F3	0.20	1.84	1.64	3.23	6.38	31.74
E50-F5	0.25	4.01	3.76	2.49	7.01	22.12
E50-F7	0.48	6.75	6.27	2.20	7.40	19.23
E50-F10	0.95	11.52	10.57	1.39	8.21	14.66

**Table 4:** Mechanical properties in terms of 100% modulus, tensile strength, elongation at break and hardness (Shore A) of ENR-50 compounded with various concentrations of FeCl_3_ at 1, 3, 5, 7 and 10 mmol and the conventional sulfur vulcanization (i.e., E50-CV).

Samples	100% Modulus (MPa)	Tensile Strength (MPa)	Elongation at Break (%)	Hardness (Shore A)
E50-CV	0.42 ± 0.03	1.63 ± 0.21	408.6 ± 56.13	30.4 ± 2.00
E50-F1	0.15 ± 0.03	0.22 ± 0.02	527.9 ± 15.12	20.11 ± 1.15
E50-F3	0.19 ± 0.02	0.49 ± 0.09	420.7 ± 19.81	22.8 ± 1.10
E50-F5	0.26 ± 0.06	0.76 ± 0.10	406.5 ± 10.93	28.7 ± 1.90
E50-F7	0.89 ± 0.18	1.89 ± 0.11	325.7 ± 20.21	44.5 ± 1.00
E50-F10	1.47 ± 0.32	4.71 ± 0.16	70.0 ± 3.00	62.5 ± 2.10

**Table 5:** Crosslinking densities of ENR-50 compounded with various concentrations of FeCl_3_ and the conventional sulfur vulcanization (i.e., E50-CV).

Sample	Crosslinking Densities (mol/m^3^)
E50-CV	99.16 ± 4.52
E50-F1	43.52 ± 8.18
E50-F3	64.45 ± 1.12
E50-F5	84.49 ± 1.98
E50-F7	112.76 ± 1.76
E50-F10	142.38 ± 0.27

**Table 6:** Degradation temperature (T_d_) of the neat ENR-50 and ENR-50 compounded with various concentrations of FeCl_3_ and the conventional sulfur vulcanization (i.e., E50-CV).

Sample	*T_d1_* (°C)	*T_d2_* (°C)	Weight Loss (%) under the Nitrogen Atmosphere	Weight Loss (%) under the Oxygen Atmosphere
Neat ENR-50	402.11	-	94.10	0.83
E50-CV	411.58	-	94.06	5.13
E50-F1	290.02	418.23	93.51	1.38
E50-F3	285.45	423.14	93.07	1.75
E50-F5	273.56	426.89	92.00	1.80
E50-F7	258.67	430.34	88.14	2.20
E50-F10	240.79	433.31	76.65	2.36

## Data Availability

Not applicable.
